# Supplementary material for: Biophysical and physiological processes causing oxygen loss from coral reefs
Source: eLife. 2019 Dec 3;8:e49114. doi: 10.7554/eLife.49114 (PMC6890468; doi:10.7554/eLife.49114)
Supplement: Supplementary file 1. — Gene abundances are provided in Figure 1—source data 1. [file elife-49114-supp1.docx]

**Supplementary Table** **1.** List of genes encoding rate-limiting enzymes participating in central carbon metabolism, reactive oxygen species detoxification and replication/translation analyzed in this study. Gene abundances are provided in Supplementary Data 1.

| **Pathway** | **Rate-limiting enzymes** | **Enzyme Commission (EC) number** |
| --- | --- | --- |
| Anaplerosis | Pyruvate carboxylase | 6.4.1.1 |
|  | Phosphoenolpyruvate carboxylase | 4.1.1.31 |
|  | Phosphoenolpyruvate carboxykinase | 4.1.1.49 and 4.1.1.32 |
|  | Glutamate dehydrogenase | 1.4.1.2 and 1.4.1.4 |
|  | Aspartate aminotransferase | 2.6.1.1 |
| Embden–Meyerhof–Parnas | Phosphofructokinase | 2.7.1.11 |
|  | Pyruvate kinase | 2.7.1.1 |
| Entner-Doudoroff | 2-keto-3-deoxygluconate-6-phosphate (KDPG) aldolase | 4.1.2.14 |
| Pentose Phosphate | 6-phosphogluconate dehydrogenase | 1.1.1.44 |
| Reactions fueling Entner-Doudoroff and Pentose Phosphate | Glucose 6P dehydrogenase | 1.1.1.49 |
|  | Glucose dehydrogenase | 1.1.1.47 |
|  | Gluconate kinase | 2.7.1.12 |
| Krebs Cycle (oxidative) | Pyruvate dehydrogenase complex (E1) | 1.2.4.1 |
|  | Oxoglutarate dehydrogenase | 1.2.4.2 |
|  | Isocitrate dehydrogenase | 1.1.1.41 and 1.1.1.42 |
| Glyoxylate cycle | Malate synthase | 2.3.3.9 |
|  | Isocitrate lyase | 4.1.3.1 |
| ROS detoxification | Superoxide dismutase | 1.15.1.1 |
|  | Glutathione reductase | 1.8.1.7 |
| Transcription | rpoB | RNA polymerase |
